# Don't worry, be happy: cross-sectional associations between physical activity and happiness in 15 European countries

**DOI:** 10.1186/s12889-015-1391-4

**Published:** 2015-01-31

**Authors:** Justin Richards, Xiaoxiao Jiang, Paul Kelly, Josephine Chau, Adrian Bauman, Ding Ding

**Affiliations:** School of Public Health, University of Sydney, Sydney, Australia; School of Public Health, Fudan University, Shanghai, China; Moray House School of Education, University of Edinburgh, Edinburgh, UK

**Keywords:** Physical activity, Exercise, Mental health, Happiness, Well-being

## Abstract

**Background:**

Mental health disorders are major contributors to the global burden of disease and their inverse relationship with physical activity is widely accepted. However, research on the association between physical activity and positive mental health outcomes is limited. Happiness is an example of a positive construct of mental health that may be promoted by physical activity and could increase resilience to emotional perturbations. The aim of this study is to use a large multi-country dataset to assess the association of happiness with physical activity volume and its specificity to intensity and/or activity domain.

**Methods:**

We analysed Eurobarometer 2002 data from 15 countries (n = 11,637). This comprised one question assessing self-reported happiness on a six point scale (dichotomised: happy/unhappy) and physical activity data collected using the IPAQ-short (i.e. walking, moderate, vigorous) and four domain specific items (i.e. domestic, leisure, transport, vocation). Logistic regression was used to examine the association between happiness and physical activity volume adjusted for sex, age, country, general health, relationship status, employment and education. Analyses of intensity and domain specificity were assessed by logistic regression adjusted for the same covariates and physical activity volume.

**Results:**

When compared to inactive people, there was a positive dose–response association between physical activity volume and happiness (highly active: OR = 1.52 [1.28-1.80]; sufficiently active: OR = 1.29 [1.11-1.49]; insufficiently active: OR = 1.20 [1.03-1.39]). There were small positive associations with happiness for walking (OR = 1.02 [1.00-1.03]) and vigorous-intensity physical activity (OR = 1.03 [1.01-1.05). Moderate-intensity physical activity was not associated with happiness (OR = 1.01 [0.99-1.03]). The strongest domain specific associations with happiness were found for “a lot” of domestic (OR = 1.42 [1.20-1.68]) and “some” vocational (OR = 1.33 [1.08-1.64]) physical activity. Happiness was also associated with “a lot” of leisure physical activity (OR = 1.15 [1.02-1.30]), but there were no significant associations for the transport domain.

**Conclusions:**

Increasing physical activity volume was associated with higher levels of happiness. Although the influence of physical activity intensity appeared minimal, the association with happiness was domain specific and was strongest for “a lot” of domestic and/or “some” vocational physical activity. Future studies to establish causation are indicated and may prompt changes in how physical activity for improving mental health is promoted.

## Background

The global burden of mental and behavioural disorders is currently estimated to account for 22.9% of years lived with disability, which is higher than any other disease category [1]. Previous research has predominantly focused on preventive and curative approaches to negative mental health disorders that are endemic in modern society [2]. It is now widely accepted that engaging in physical activity positively influences depression and anxiety across the lifespan [3-6]. However, there is less evidence regarding the relationship between physical activity and positive mental health constructs that may subsequently increase resilience to emotional perturbations [4,7-9]. This paucity of research is evident in physical activity behaviour change messages that have previously focused on disease prevention rather than the promotion of positive mental health outcomes (e.g. physical activity to prevent depression rather than to promote happiness). It is thought that examining positive components of mental well-being, such as happiness, may identify new opportunities to apply health promotion models to physical activity interventions and improve mental health outcomes [10].

Despite a range of definitions for the construct of happiness, there is general consensus that it comprises positive mental or emotional (affective) states of enjoyment and contentment [9-11]. It appears that the promotion of happiness is emerging as a public health priority in several countries globally. This is evident in a recent report published by the World Health Organisation (WHO) Bulletin that indicated countries such as the UK, France and Canada are following Bhutan’s example and considering national happiness indexes to complement existing population measures of development such as Gross Domestic Product (GDP) [11]. This represents a clear departure from measuring economic growth as the sole indicator of national prosperity and has stimulated debate on how happiness can be facilitated [12,13]. Lyubomirsky et al. (2005) described three general predictors of happiness: (i) life circumstances and demographics; (ii) traits and dispositions; (iii) intentional behaviours [12]. From a public health perspective, “intentional behaviour” is the only one of these predictors that is individually modifiable and as an example of this, physical activity has been identified as a strategy that may induce happiness [13].

Several literature reviews have described a positive association between physical activity and mental well-being, but there is a paucity of studies specific to the construct of happiness [6,7,14]. The existing evidence primarily comprises single-country cross-sectional analyses that indicate happiness is positively associated with higher volumes of physical activity [15-17]. These studies have used different methods to assess happiness and to our knowledge there are no previous multi-country analyses that have assessed the association with physical activity using consistent metrics. There is also limited evidence describing the relationship of different physical activity domains (i.e. domestic, leisure, transport, vocation) and intensities (i.e. moderate, vigorous) with happiness. This makes it difficult to target specific physical activity promotion messages to activities that may be more beneficial. Although previous analyses of population survey data from several countries indicate that physical activity for leisure appears to have a stronger association with mental health than other domains, these studies have not assessed the construct of happiness [18-20]. It also appears that physical activity intensity may be an important moderating factor for various mental health outcomes, but existing recommendations focus on physical outcomes and there is currently insufficient evidence to target these towards improving emotional well-being [9,21-23].

In this paper, our primary objective is to use a large multi-country European dataset to examine the association between self-reported physical activity and happiness. Our secondary objective is to analyse the domain- and intensity-specific associations of physical activity with happiness. We intend the results to inform future research and policy on targeting physical activity interventions to promote happiness and other positive constructs of mental health.

## Methods

### Study sample and data collection procedures

The Eurobarometer is a multi-national survey of public opinion and social trends in the European Union conducted since the 1970s on behalf of the European Commission (http://www.gesis.org/eurobarometer). In this study, we accessed data from Eurobarometer 58.2, which was the most recent survey that collected concurrent data on physical activity and happiness (October 28 - December 8, 2002) [24]. The data is publicly available and its use for non-commercial research is authorized.

Eurobarometer 58.2 was conducted among 15,334 respondents from 15 European Union countries: Austria, Belgium, Denmark, Finland, France, Germany, Greece, Ireland, Italy, Luxembourg, Netherlands, Portugal, Spain, Sweden, United Kingdom. All European Union citizens residing in those countries (nationals and non-nationals) who were at least 15 years old were eligible to participate. Sample sizes within countries ranged from n = 602 (Luxembourg) to n = 2,042 (Germany) and the overall response rate was 54.6%.

Participants in the Eurobarometer 58.2 survey were sampled via a multi-stage design. First, primary sampling units (PSU) were selected from each European statistical administrative region in participating countries, with the sampling probability proportional to population size and stratified by urbanisation. Second, addresses were randomly selected within each PSU, and then one respondent was randomly selected per household. All necessary informed consent was obtained for each respondent and they were interviewed face-to-face in their homes in their national language. The data were collected by the European Opinion Research Group, which is a consortium of marketing and public opinion research agencies. Further details of Eurobarometer methods are available online (http://ec.europa.eu/public_opinion/description_en.htm).

### Measures

The Eurobarometer 58.2 survey categories for socio-demographic data about the participants were used for: sex (male, female); age (15–24, 25–39, 40–54, 55 years and older); age when finished full-time education (≤15 years, 16–19 years, ≥20 years, still studying); self-reported general state of health (very good, good, fair, bad/very bad); employment status (self-employed, employed, not working). We recoded the relationship status responses into a dichotomized variable based on current cohabitation with a partner (currently cohabitating: “married”, “remarried”, “unmarried, currently living with partner”; not currently cohabitating: “unmarried, having never lived with a partner”, “unmarried, having previously lived with a partner but on my own”, “divorced”, “separated” and “widowed”).

The measurement of happiness was adopted from a single question from the SF-36 survey: “In the past month, have you felt happy?” [25]. There were six response options that we recoded into a dichotomized variable to differentiate between those who provided a positive vs. negative response (YES: “all the time”, “very often”, “often”; NO: “rarely”, “very rarely”, “never”).

Physical activity was assessed by adapting questions from the short form of the International Physical Activity Questionnaire (IPAQ-short) into a multiple-choice format as presented in Table 1 [26]. The IPAQ-short had test-retest reliability (Spearman’s rho = 0.76) and criterion validity (Spearman’s rho = 0.30) that was comparable to other self-report measures [27]. Participants were asked to report the number of days in the past week and the total time per day (hours/minutes) of walking, moderate-intensity and vigorous-intensity physical activity in bouts of at least 10 minutes. We calculated physical activity volume as the sum of the three activities with vigorous physical activity weighted by two to account for higher energy expenditure. Participants were then classified based on the global physical activity recommendations as “inactive” (0–9 minutes of physical activity), “insufficiently active” (10–149 min), “sufficiently active” (150–299 min), and “very active” (300+ min). We also calculated the independent amounts of walking, moderate-intensity and vigorous-intensity physical activity for subsequent analysis of intensity specific associations with happiness.

**Table 1:** **Adaptation of the of the IPAQ-short questions used in Eurobarmoeter 58.2**

Q50.	In the last seven days, on how many days did you do vigorous physical activities like lifting heavy things, digging, aerobics or fast cycling?
	None / 1 / 2 / 3 / 4 / 5 / 6 / 7 / Don’t know
Q51.	On days when you do vigorous physical activities, how much time do you usually spend at it (hours)?
	<1.0 / 1.0–1.5 / 1.5-2.5 / 2.5-3.5 / 3.5-4.5 / >4.5 / Don’t know
Q52.	In the last seven days, on how many days did you do moderate physical activities like carrying light loads, cycling at a normal pace or doubles tennis? Please do not include walking.
	None / 1 / 2 / 3 / 4 / 5 / 6 / 7 / Don’t know
Q53.	On days when you do moderate physical activities, how much time do you usually spend at it (hours)?
	<1.0 / 1.0–1.5 / 1.5-2.5 / 2.5-3.5 / 3.5-4.5 / >4.5 / Don’t know
Q54.	In the last seven days, on how many days did you walk for at least ten minutes at a time?
	None / 1 / 2 / 3 / 4 / 5 / 6 / 7 / Don’t know
Q55.	On days when you walk for at least ten minutes at a time, how much time do you usually spend walking (hours)?
	<1.0 / 1.0–1.5 / 1.5-2.5 / 2.5-3.5 / 3.5-4.5 / >4.5 / Don’t know

The Eurobarometer 58.2 survey included four additional questions to assess physical activity in various domains (i.e. vocation, transport, domestic and leisure). The participants were asked: “In the last seven days, how much physical activity did you get: 1) at work; 2) when moving from place to place; 3) doing work in and around your house (including housework, gardening, general maintenance, or caring for your family); 4) for recreation, sport and leisure-time activities?” There were three response options: “a lot”, “some” or “little or none”. These data were used to assess domain specificity of the association between physical activity and happiness.

### Statistical analysis and reporting

Data analyses were performed using IBM SPSS19.0. Participants who did not have complete physical activity data (n = 3,607) and an additional 90 individuals who reported they were “severely restricted to physical activity” were excluded from the final analytical sample. Reported happiness was presented according to socio-demographic characteristics and bivariate associations were examined using a Pearson’s chi-square test. We ran logistic regressions to examine the crude and adjusted associations of happiness with: 1) physical activity volume; 2) physical activity intensity; 3) physical activity domain. The model for physical activity volume was adjusted for sex, age group, country, general state of health, relationship status, employment status and age when finished full-time education. The model for physical activity intensity included time spent engaging in walking, moderate-intensity and vigorous-intensity physical activity as independent variables, adjusted for all of the covariates described above. Since previous studies have identified sex differences for the association between physical activity intensity and mental health, we tested these interactions and conducted stratified analysis for any significant findings [21]. The associations between domains of physical activity and happiness were tested in separate models adjusted for physical activity volume in addition to all covariates. The model for vocational physical activity only included those currently employed (n = 5,816). Statistical significance was assessed at the levels of p < 0.05, p < 0.01 and p < 0.001.

## Results

### Descriptive statistics

A total of 11,637 participants with complete data from 15 European Union countries (75.9% of the original sample) were included in the current study. Descriptive statistics on proportion of feeling happy by country and relevant variables are presented in Table 2. Overall, 82.9% of the participants reported feeling happy (all the time, very often or often) in the past month. Significant differences were observed across socio-demographic factors and by country. Italians (72.4%) and Germans (73.8%) reported the lowest proportions of feeling happy and Dutch (91.1%) and Irish (90.4%) reported the highest proportions of feeling happy. The participants were more likely to report feeling happy if they were male, of younger age groups (i.e. below 40 years), in good health, currently in a relationship and working. Besides a positive association between educational attainment and feeling happy, those who were still studying were the most likely to report being happy. There was a significant and positive association between physical activity participation level and happiness, with nearly 86% of those who were very active reporting feeling happy.

**Table 2:** **Descriptive table of happiness by variables of interest (n = 11,637)**

	**n (%)**	**% Feeling happy**	**p-value**
**Sex**			
Male	5622 (48.3)	85.1	<0.001
Female	6015 (51.7)	80.9	
**Age group**			
15-24	2009 (17.3)	87.9	<0.001
25-39	3354 (28.8)	85.3	
40-54	2713 (23.3)	81.6	
≥55	3561 (30.6)	78.8	
**Country**			
Austria	713 (6.1)	81.0	<0.001
Belgium	777 (6.7)	86.1	
Denmark	721 (6.2)	83.9	
Finland	814 (7.0)	86.4	
France	818 (7.0)	77.3	
Germany	1327 (11.4)	73.8	
Greece	731 (6.3)	82.4	
Ireland	859 (7.4)	90.4	
Italy	792 (6.8)	72.4	
Luxemburg	326 (2.8)	79.6	
Netherlands	619 (5.3)	91.1	
Portugal	608 (5.2)	81.1	
Spain	759 (6.5)	88.1	
Sweden	769 (6.6)	86.8	
United Kingdom	1004 (8.6)	87.4	
**General state of health**			
Bad/very bad	371 (3.2)	41.8	<0.001
Fair	2632 (22.6)	69.9	
Good	5196 (44.7)	85.9	
Very good	3423 (29.5)	92.7	
**Relationship status**			
Living with someone	6887 (59.3)	86.2	<0.001
Not living with someone	4719 (40.7)	78.2	
**Employment status**			
Non-active	5685 (48.9)	80.9	<0.001
Self-employed	852 (7.3)	86.1	
Employed	5100 (43.8)	84.7	
**Age when finished full-time education**			
≤14 years	1908 (16.4)	77.2	<0.001
15-21 years	6424 (55.2)	82.4	
≥22 years	1920 (16.5)	85.9	
Still studying	1385 (11.9)	88.8	
**Physical activity level**			
Inactive	1026 (8.8)	73.0	<0.001
Insufficiently active	1709 (14.7)	78.3	
Sufficiently active	1708 (14.7)	82.0	
Very active	7194 (61.8)	85.7	

Chi-square test for significance between distributions among variables of interest.

### Physical activity and happiness

The crude and adjusted odds ratios of the association of feeling happy with physical activity volume, intensity and domain are presented in Table 3.

**Table 3:** **Multiple binary logistic regression of probability of being happy (n = 11,637)** ^**1**^

	**Unadjusted OR**	**Adjusted OR**
**Physical activity volume** (reference: inactive)^2^		
Insufficient physical activity	1.34***(1.12-1.60)	1.20*(1.03-1.39)
Sufficient physical activity	1.68***(1.40-2.02)	1.29**(1.11-1.49)
Very active	2.21***(1.89-2.58)	1.52***(1.28-1.80)
**Intensity specific physical activity** (unit: hours)^3^		
Vigorous-intensity	1.07***(1.05-1.09)	1.03*(1.01-1.05)
Moderate-intensity	1.04***(1.03-1.06)	1.01(0.99-1.03)
Walking	1.03***(1.02-1.05)	1.02*(1.00-1.03)
**Physical activity domain** ^**4**^		
**Vocation physical activity level** (reference: little/none)^5^		
Some	1.38***(1.18-1.61)	1.39**(1.15-1.67)
A lot	1.37***(1.18-1.60)	1.33**(1.08-1.64)
**Transport physical activity level** (reference: little/none)		
Some	1.15***(1.05-1.27)	1.03(0.91-1.16)
A lot	1.49***(1.29-1.72)	1.07(0.89-1.29)
**Domestic physical activity level** (reference: little/none)		
Some	1.34***(1.21-1.48)	1.36***(1.20-1.53)
A lot	1.52***(1.34-1.72)	1.42***(1.20-1.68)
**Leisure physical activity level** (reference: little/none)		
Some	1.65***(1.50-1.81)	1.14(0.95-1.36)
A lot	2.24***(1.94-2.59)	1.15*(1.02-1.30)

^1^All participants who had no physical activity data or were “severely restricted to physical activities” were excluded.

^2^Adjusted for sex, age, country, general health, relationship status, employment status, age finished full-time education.

^3^Adjusted for sex, age, country, general health, relationship status, employment status, age finished full-time education, other categories of physical activity intensity.

^4^Adjusted in separate models for sex, age, country, general health, relationship status, employment status, age finished full-time education, physical activity volume.

^5^Only participants reporting current employment were included in the vocational analysis (n = 5,816).

Statistical significance: *p < 0.05, **p < 0.01, ***p < 0.001.

A positive dose–response relationship was found for physical activity volume and happiness. Compared to inactive participants, the adjusted odds of being happy was 20% higher for people insufficiently active and this increased to 29% and 52% higher for those sufficiently and very active respectively.

All crude odds ratios for the association between happiness and different physical activity intensities (i.e. walking, moderate, vigorous) were small, but statistically significant. These associations only remained statistically significant for walking and vigorous-intensity physical activity in the adjusted model. The adjusted results indicate that the odds of being happy were 2% higher for each additional weekly hour of walking and 3% higher for each additional hour of vigorous physical activity. There were no significant interactions with sex for our analyses of walking [p = 0.21] or moderate intensity physical activity [p = 0.20]. However, we found a significant interaction with sex for our vigorous physical activity result [p = 0.002]. The sex stratified analyses found a significant association between vigorous physical activity and happiness for females [OR = 1.07, 95% CI = 1.03-1.11], but not for males [OR = 1.01, 95% CI = 0.98-1.03].

There was a statistically significant crude association between happiness and doing “some” or “a lot” of physical activity in each domain (i.e. vocation, transport, domestic and leisure). These associations remained statistically significant for the vocational and domestic domains after adjusting for socio-demographic variables and physical activity volume. Domestic physical activity appeared to have the strongest association with happiness in the adjusted models. Compared with people who reported “little or no” domestic physical activity, those who reported “some” had 36% higher odds of feeling happy and this further increased to 42% for those who reported “a lot”. In contrast, compared with people who reported “little or no” vocational physical activity, those who reported “some” had 39% higher odds of feeling happy and this decreased to 33% for those who reported “a lot”. Participants who reported “some” leisure physical activity were not significantly different to those who reported “little or none”, but those doing “a lot” had 15% higher odds of feeling happy. The amount of physical activity in the transport domain was not significantly associated with feeling happy in the adjusted model.

## Discussion

Findings from our study indicate that increasing volumes of physical activity are associated with higher levels of happiness. The intensity of physical activity appears to be of minimal importance. However, the association with happiness was domain specific and our results indicate it was strongest for people who engaged in “some” vocational and/or “a lot” of domestic physical activity.

To our knowledge, this paper presents the first multi-country analyses of the association between physical activity and happiness. Our results for the association between physical activity volume and happiness are similar to previous cross-sectional analyses completed in several healthy populations. Kye et al. (2014) assessed a random sample of 1,530 people in Korea aged 30–69 years and found that those who exercised for 30 minutes at least five times per week were more likely to be happy [17]. Similarly, a study of 3,461 university students aged 17–24 years in Chile found that those who engaged in daily physical activity were more likely to be happy [16]. Moljord et al. (2011) examined 1,508 adolescents aged 13–18 years in Norway and found that those who participated more frequently in physical activity were significantly happier [15]. Secondary analysis of four population surveys from the USA and Canada that included people aged 10 years and older also demonstrated an association between physical activity and positive mood [19]. This collection of papers suggests a global phenomenon that extends across the lifespan and aligns with the broader evidence for other positive constructs of mental health [6,7,14,28].

However, several authors remain sceptical about the association between physical activity and happiness. Blacklock et al. (2007) suggested that the contribution of physical activity to happiness might be minor compared to other demographic and lifestyle factors such as education, income and companionship [29]. Similarly, a longitudinal co-twin study conducted in the Netherlands concluded that an underlying environmental or genetic confounding factor may be positively influencing both physical activity participation and happiness [30]. This ongoing conjecture warrants further prospective intervention studies to establish the relationship between physical activity and happiness.

Our finding that only walking and vigorous-intensity physical activity had small associations with happiness adds to ongoing debate in the literature regarding intensity-specificity. Although a previous review of exercise and mood state suggested that moderate-intensity physical activity is the most beneficial, it is thought that this may vary according to the personal preference of each individual [22]. An assessment of 6,803 Belgian adults suggested that sex may moderate the association between physical activity intensity and several constructs of mental health [21]. However, our results contrasted with the findings of this previous study, which suggested better mental health outcomes were most strongly associated with physical activity at a vigorous intensity for men and at a moderate intensity or walking for women [21]. Although it is important to note that previous research has focused on broader constructs of mental health than happiness, this ongoing uncertainty suggests further sex specific investigation is necessary.

In contrast, we found several stronger associations between different domains of physical activity and happiness. The results for the vocational domain suggest that optimal happiness is associated with performing some physical activity at work, but not a lot. This concurs with existing evidence for the deleterious effects on mental health of both highly sedentary lifestyles and work that is primarily manual labour [31,32]. There appears to be a “happy medium” for vocational physical activity and further research is indicated to identify the most effective ways of facilitating this in different workplaces.

Our results for the transport domain also concur with existing data. The Taking Part Survey included more than 24,000 British adults and demonstrated that happiness is associated with participation in recreational sport, but not cycling for utilitarian purposes (e.g. transport to work) [33]. This may be explained by concerns about safety and the underlying recreational versus utilitarian motives for physical activity in the transport domain (i.e. commuting is often a necessary process that “must be done” and is inherently stressful) [9,20,33]. Consequently, perhaps future research on the relationship between active transport and happiness should focus on comparisons with motorised commuting, rather than other physical activity domains.

Contrary to our results suggesting that domestic physical activity has the strongest association with happiness, the existing evidence indicates that the leisure domain may be more important. Secondary analysis of the 1978 Canadian Health Survey found that women were considerably happier when engaged in recreational activities only than when domestic chores were part of their physical activity mix [19]. Our contrasting findings may reflect changes that have occurred between 1978 and 2002 in social perceptions of domestic activities and gender roles and/or variation in cultural norms between Canada and Europe. It is also likely that further changes have occurred since the 2002 Eurobarometer data was collected and this may limit the relevance of our results to current associations between physical activity and happiness in the participating countries. However, more recent data from 19,842 adult participants in the 2009 Scottish Health Survey also indicated an inverse association with negative constructs of mental health that was stronger for leisure than domestic physical activity [18]. Similarly, a Belgian study of 1,919 adults demonstrated variation according to sex and other socio-demographic factors, but also indicated that psychological distress was associated with recreational sport participation and inversely associated with housework [20].

We hypothesise that the strong association between happiness and domestic physical activity in the 2002 Eurobarometer data was because the measurement tool explicitly referred to tasks that are often recreational (i.e. gardening) and promote self-worth (i.e. caring for family) [24]. As described by Aszatalos (2012), these are typical characteristics of “mindful” physical activity that is driven by a positive underlying motivation and is more likely to be associated with better mental health outcomes [9]. In contrast, the domestic domains of the Canadian Health Survey focussed on “chores”, the Scottish Health Survey referred to “heavy housework/manual and gardening work” and the previously described Belgian study also assessed “housework” [18-20]. These descriptions invoke different connotations to the terms used in the Eurobarometer survey and demonstrate how the heterogeneous adaptation of physical activity measures in different settings may limit comparisons between studies.

Methods for assessing happiness have also been subject to ongoing debate and in 2011 there were more than 1,200 measurement items listed on the World Database of Happiness [34]. The question we used was derived from the widely utilised SF-36 Health Survey, but had not been validated in isolation. Despite continued conjecture about the use of a single-item self-report measure of happiness, the early work of Fordyce (1977) demonstrating this to be the most psychometrically valid approach is yet to be refuted [35]. However, there is evidence to suggest that the construct of happiness is inconsistent across languages and this may have implications on the validity of the findings in this multi-country study [34]. Furthermore, the relationship with mental health outcomes may vary depending on whether physical activity is measured subjectively (e.g. self-report) or objectively (e.g. accelerometer) [36,37]. The limitations of self-report data for physical activity are well documented and although this has not been examined when assessing associations with positive constructs of mental health, it weakens our conclusions and may be an important consideration in future studies.

Another limitation of our study is the cross-sectional design. There has been limited research to establish the existence and direction of a causal relationship between physical activity and happiness. Wang et al. (2012) linked data from consecutive National Population Health Surveys in Canada and found that leisure physical activity was associated with a decreased likelihood of becoming unhappy and may help maintain happiness over time [38]. Similar conclusions were drawn from a survey of 438 Norwegian adults that found that retrospectively reported adolescent exercise levels were correlated with positive mood and happiness in adulthood [39]. However, the relationship between adolescent physical activity and adult happiness was no longer significant after adjustment for adult physical activity levels [39]. Consequently, further investigation into the direction of causation and the longitudinal relationship between physical activity and happiness is warranted.

## Conclusions

In conclusion, our results demonstrate for the first time that happiness is associated with physical activity participation across multiple countries. This study adds impetus to a potential paradigm shift for physical activity and mental health towards promoting positive outcomes that may be more appealing to the population and increase resilience to mental health disorders. However, further longitudinal research is indicated to dissect the direction of causation and the specificity of physical activity domain and intensity for different mental health constructs.

## Abbreviations

GDP: Gross domestic product
IPAQ: International physical activity questionnaire
OR: Odds ratio
PSU: Primary sampling unit
WHO: World Health Organisation
